# In Situ Immobilization on the Silica Gel Surface and Adsorption Capacity of Poly[*N*-(4-carboxyphenyl)methacrylamide] on Toxic Metal Ions

**DOI:** 10.1186/s11671-017-2066-0

**Published:** 2017-04-27

**Authors:** Elina Yanovska, Irina Savchenko, Dariusz Sternik, Olga Kychkiruk, Lidiya Ol’khovik, Iana Buriachenko

**Affiliations:** 1 Taras Shevchenko National University of Kyiv, 12 L. Tolstogo St., 01033 Kyiv, Ukraine; 2 Maria Curie-Sklodowska University, pl. Maria Curie-Sklodowskiej 3, 20-031 Lublin, Poland; 3 Ivan Franko Zhytomyr State University, 40 Velyka Berdychivska St., 10008 Zhytomyr, Ukraine

**Keywords:** Poly[*N*-(4-carboxyphenyl)methacrylamide], Modified silica, In situ immobilization, Metal complexes, Adsorption properties

## Abstract

In situ immobilization of poly[*N*-(4-carboxyphenyl)methacrylamide] has been performed on silica gel surface. Infrared (IR) and mass spectroscopies as well as thermogravimetry (TG) analysis have been used to elucidate the structure of immobilized polymer. An adsorption capacity of the synthesized composite towards Cu(II), Pb(II), Mn(II), Fe(III), Co(II), and Ni(II) ions has been estimated. Adsorption activity to microquantities of Pb(II), Cu(II), and Ni(II) in a neutral aqueous medium has been observed.

## Background

Complex forming chemically modified silica are well known as efficient adsorbents are widely used in modern hybrid and combined methods of analytical chemistry, at the development of new treatment technologies. Increase of the adsorption capacity of transition metal ions on the surface of silica is advisable to consolidate macromolecules with complexing groups (polyaniline, polyacrylic acid, polyionenes etc.) [1–7].

Binding capable of complexing polymers on the surface of silica and other inorganic oxide matrices can be made by adsorption of polymers [8] and formation of chemical bonds with surface sites [1, 9].

However, the promising one-step synthesis of complexing composite materials, which leads to even distribution of active complexing groups in the polymer immobilized on the surface of the inorganic matrix, is direct (in situ) formation of the polymer layer in the presence of particles of inorganic matrix [10–12].

The advantages of in situ polymer-modified solid surfaces compared with the physical adsorption of pre-synthesized polymers on the inorganic carrier include the following:

- The optimal (both energy and geometric) location of polymer on a solid surface, which enhances the fixing of polymer on the inorganic carrier and therefore creates additional opportunities for the regeneration processes after repeated use of the composite material as the adsorbent
- The possibility of self-organization of polymer chains in supramolecular structures on the surface of the inorganic carrier, which increases the adsorption capacity of organic-mineral composite [13]

Compared with chemical fixing of pre-synthesized polymers, in situ immobilization is characterized by the absence on the solid surface of carrier, the residues of monomolecular compounds that are used as a bridging groups between the polymer and inorganic matrix.

All these lead to expansion of the range of immobilized polymer adsorption ability and improve its adsorption capacity.

This work is dedicated in situ immobilization of poly[*N*-(4-carboxyphenyl)methacrylamide] on the silica gel surface, the study of surface morphology of modified silica gel and adsorption properties of the synthesized composite material on ions Cu(II), Pb(II), Mn(II), Fe(III), Co(II), and Ni(II).

## Methods

The polymerization of *N*-(4-carboxyphenyl)methacrylamide in the presence of silica (fraction of particles with a diameter of 0.1–0.2 mm, specific surface 428 m^2^/g, Merck) has been carried out under the argon atmosphere. A solution of 6.72 g *N*-(4-carboxyphenyl)methacrylamide and 0.0672 g 2,2′-azobisisobutyronitrile (AIBN) in 60 ml tetrahydrofuran (THF) were poured into a flask containing 15 g of silica gel. When argon blowing was finished after 15 min, the reaction mixture was heated to 78 °C; polymerization continued for 5 h with stirring. The reaction was stopped by cooling the reaction mixture. The resulting suspension was poured into a porcelain cup and left overnight to evaporate the solvent; the synthesized composite was washed three times with isopropyl alcohol, filtered, and air-dried for 24 h at room temperature.

IR spectra of the output silica gel, monomer, and composite have been recorded with Thermo Nicolet Nexus FTIR (USA) infrared spectrophotometer with Fourier transformation. The amount of polymer on the surface of silica gel was evaluated by thermogravimetric analysis results obtained with simultaneous TG/DTA analyzer Shimadzu DTG-60H (Japan) with computer registration of data in the 15–1000 °C temperature range. The heating rate of samples is 10 °C/min.

Thermal analysis was carried out on a STA 449 Jupiter F1, Netzsch (Germany), under the following operational conditions: heating rate of 10 °C min^−1^, a dynamic atmosphere of synthetic air (50 mL min^−1^), temperature range of 30–950 °C, sample mass ~18 mg, and sensor thermocouple type S TG-DSC. As a reference, empty Al_2_O_3_ crucible was used. The gaseous products emitted during decomposition of materials were analyzed by FTIR spectrometer Brucker (Germany) and by QMS 403C Aeölos (Germany) coupling on-line to STA instrument. The QMS data were gathered in the range from 10 to 160 amu. The FTIR spectra were recorded in the spectral range of 600–4000 cm^−1^ with 16 scans per spectrum at a resolution of 4 cm^−1^.

To study the parameters of the surface of the synthesized composite, the BET method (low-temperature nitrogen adsorption-desorption) at the boiling point of liquid nitrogen is used with ASAP 2420 V1.01 Sorbtometer (Micromeritics, USA). Before measuring, the samples are degassed at 60 °C. As a result of computer processing of nitrogen adsorption-desorption isotherms, the surface area of composite and pore diameter distribution are determined.

Adsorption characteristics of synthesized composite material on Cu(II), Pb(II), Mn(II), Fe(III), Ni(II), and Cd(II) ions are investigated in static mode. Thus, 0.1 g of the composite contacts 25–100 ml working solutions of nitrates of the corresponding metals while constantly stirred with a mechanical vibrator at room temperature.

Determination of the equilibrium concentration of the metals is carried out by atomic absorption using a flaming atomic absorption spectrophotometer Saturn (Ukraine) in a “air-propane-butane” flame mixture.

The degree of adsorption (*R*) is calculated using the formula

1 $$ R = \left({m}_{\mathrm{ads}}/{m}_o\right) \cdot p\ 100\% = \left({m}_o\hbox{--} m\right)/{m}_o \cdot p\ 100\% $$

where *m*_o_ is the mass of metal in the output solution, *m*_ads_ is the mass of adsorbed metal, and *m* is the mass of metal in the equilibrium solution after adsorption, which is calculated as *m* = [*M*] · *V*, where [*M*] is the equilibrium concentration of metal and *V* is the volume of equilibrium solution.

Working nitrate solutions of Cu(II), Pb(II), Mn(II), Fe(III), Ni(II), and Cd(II) are prepared with the sets of “standard sample solutions” of these salts on 1 M HNO_3_ background (produced by A.V. Bogatsky Institute in Odesa) with concentrations of 1 and 10 mg/ml. To create proper pH environment, standard buffer solutions are used (ISO 8135: 2009, manufacturer—JSC “Kyiv Plant RIAP”).

Research of the adsorption capacity of the synthesized composite to the listed transition metal ions includes the determination of the optimal range of pH-adsorption medium, the determination of the required contact time to achieve phase adsorption equilibrium in static mode and building of sorption isotherms of the appropriate metal ions to establish the adsorption capacity.

To create an actual acidity in studies of adsorption processes, the standard buffers made from titration substance are used. pH buffer solutions are monitored by ionomer “HANNA HI 98129” with glass electrode.

## Results and Discussion

**Fig. 1:**
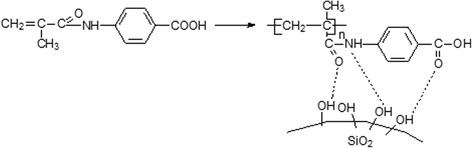
The scheme of in situ immobilization of poly[*N*-(4-carboxyphenyl)methacrylamide] on the silica surface

The chemical structure of in situ polymer immobilization *N*-(4-carboxyphenyl)methacrylamide on the silica surface could be presented in Fig. 1.

**Fig. 2:**
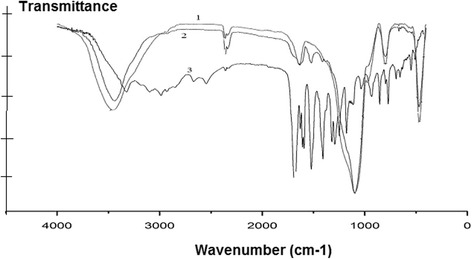
The FTIR spectra of the original silica gel (*1*) monomer (*3*) and synthesized composite (*2*)

The fact of the process of in situ polymerization and fixing of the polymer on the silica surface were confirmed by comparative analysis of IR spectra of synthesized composite, monomer, and original silica gel (Fig. 2).

As you can see from this figure, the most informative part of the infrared spectrum of the synthesized composite is the region from 1200 to 1700 cm^−1^, where there are a number of absorption bands that can be attributed to the stretching vibrations υ(C = O) bonds of polymer and stretching vibration of aromatic system [14].

**Fig. 3:**
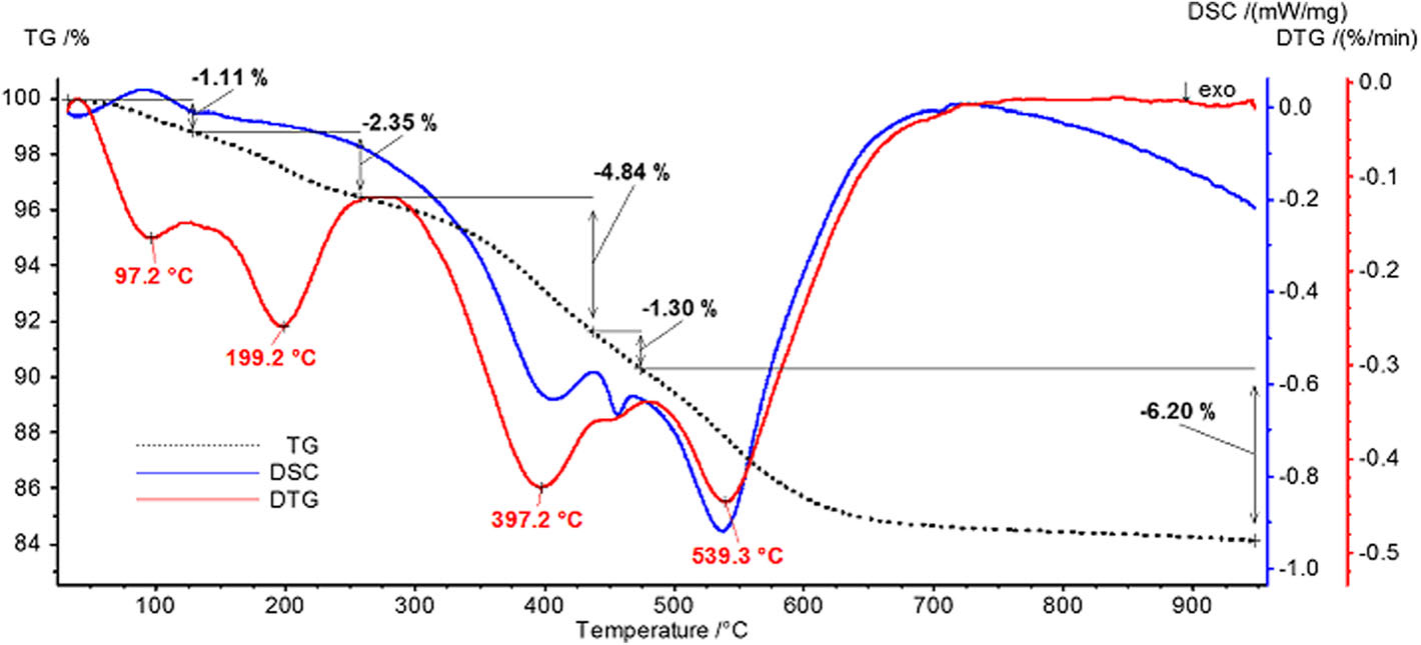
Thermogram of synthesized composite

To determine the mass of immobilized polymer, thermogravimetric analysis was performed (Fig. 3). It can be seen from the thermogram presented that most of the polymer decomposes in the temperature range from 100 to 600 °C. Approximately 16% of the composite weight is lost, which suggests that this is exactly the mass of polymer to be found on the silica gel surface.

From Fig. 3, the process of thermal destruction of immobilized polymer can be divided into four stages: stage I is in the temperature range from 100 to 250 °C, and the mass loss is 2.35%, the largest part of which is lost at 199.2 °C; stage II is in the temperature range from 250 to 420 °C, where weight loss is 4.84%, most of which is lost at 397.2 °C; stage III is in the temperature range from 420 to 480 °C, where weight loss is 1.3%; stage IV is in the temperature range from 480 to 600 °C, with a loss of 6.2% by weight, most of which occurs at 530 °C.

**Fig. 4:**
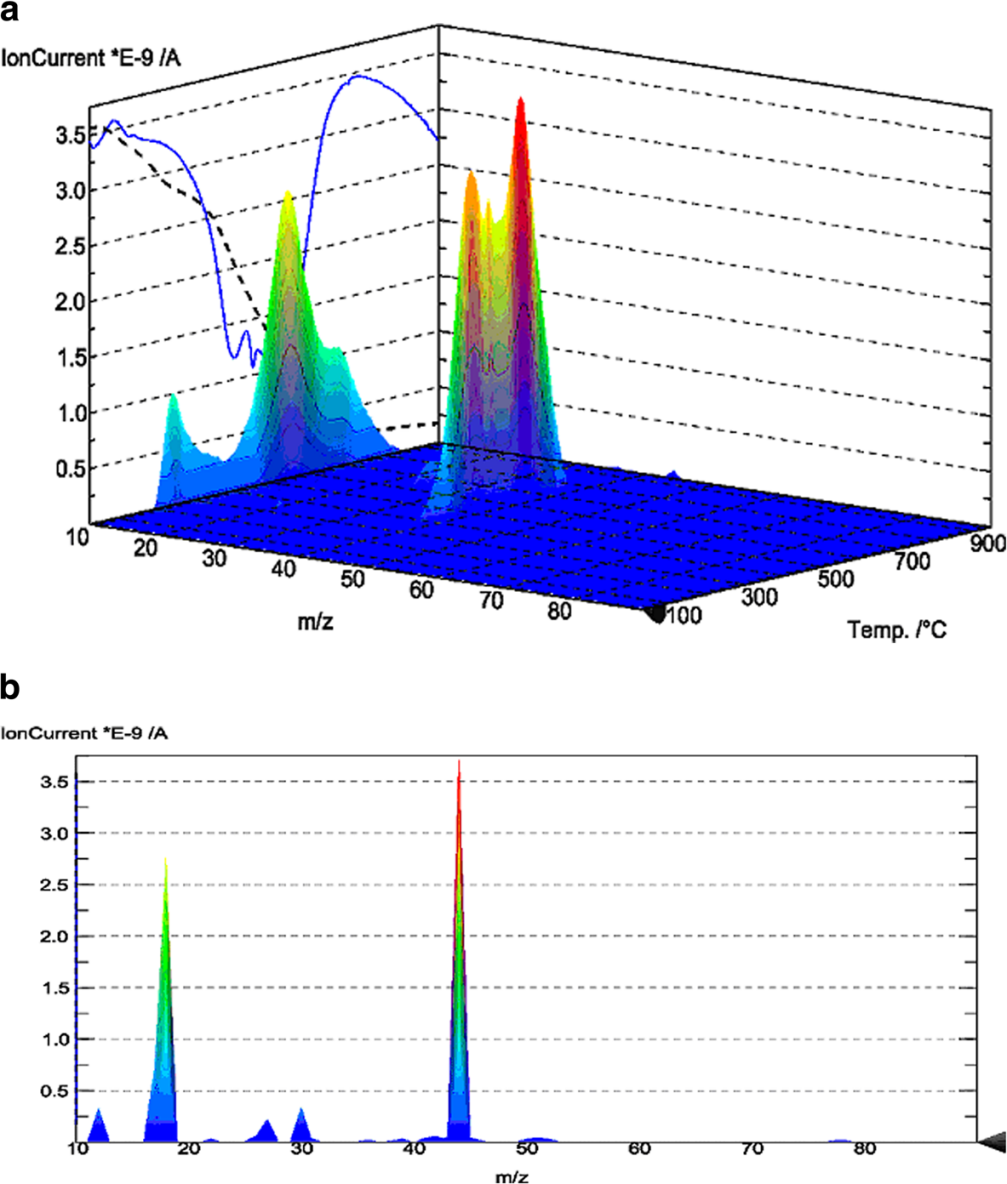
**a** TG-MS-3D of synthesized composite. **b** 2D mass spectrum of synthesized composite

From these figures (Fig. 4a, b), the peak of high intensity with a mass of 18 corresponds to the mass loss of water. Small peak with a mass of 28 may correspond to the loss of CO or N_2_. The peak of low intensity with a molecular weight of 30 can be attributed to the loss of the carbon chain part.

**Fig. 5:**
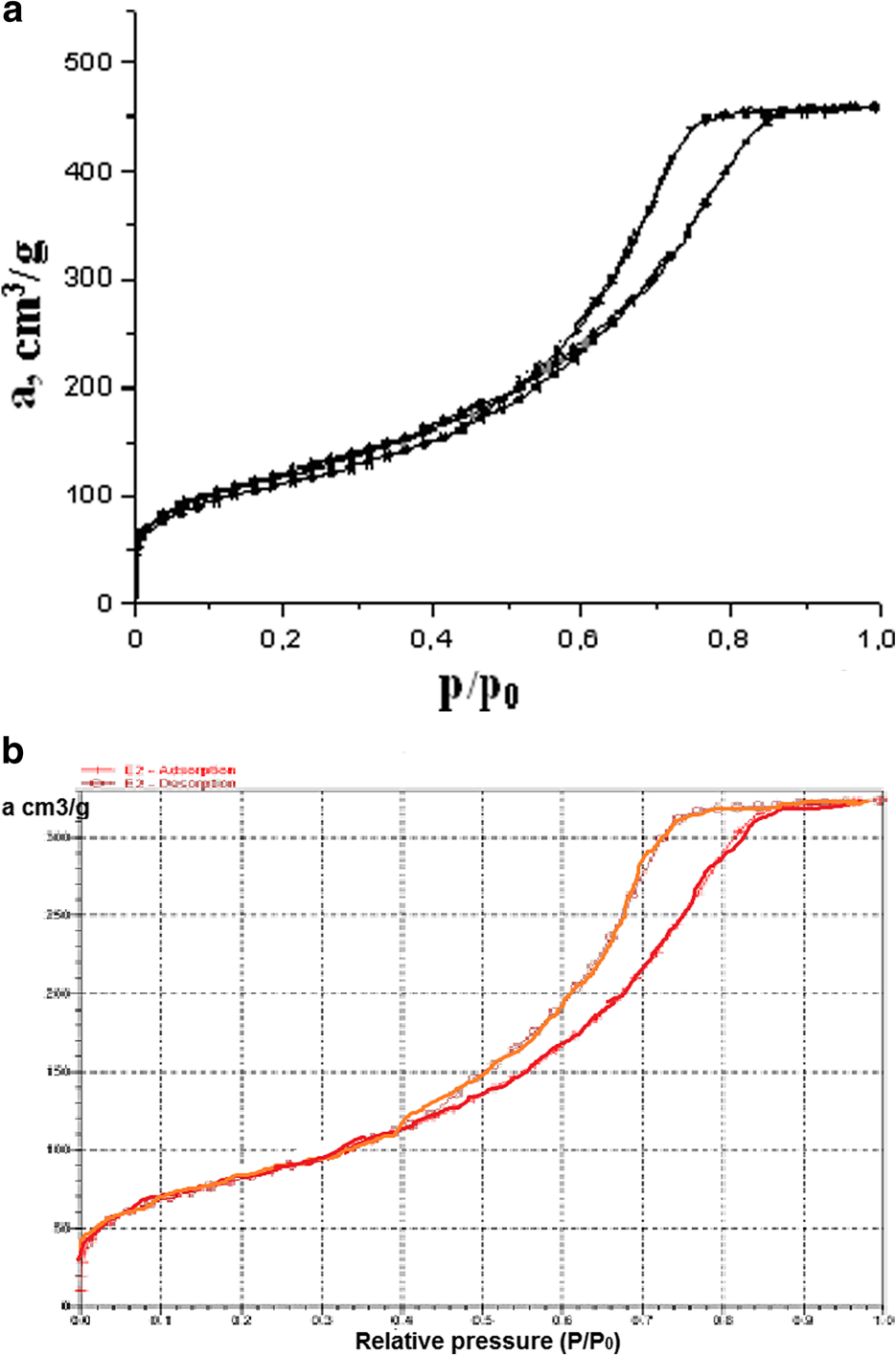
Adsorption-desorption isotherms of nitrogen of original silica **a** and composite **b**

For investigation of the morphology of the silica surface after modification by polymer, the adsorption-desorption isotherms of nitrogen of original silica gel and composite were compared (Fig. 5).

As follows from Fig. 5, obtained isotherms are similar and belong to type IV isotherms according to IUPAC classification [15]. The data indicate that immobilized polymer has virtually no effect on the structure of the surface layer of silica gel.

The value of the specific surface area of silica gel after immobilization by polymer, calculated using the method of BET, is 299 m^2^/g.

**Fig. 6:**
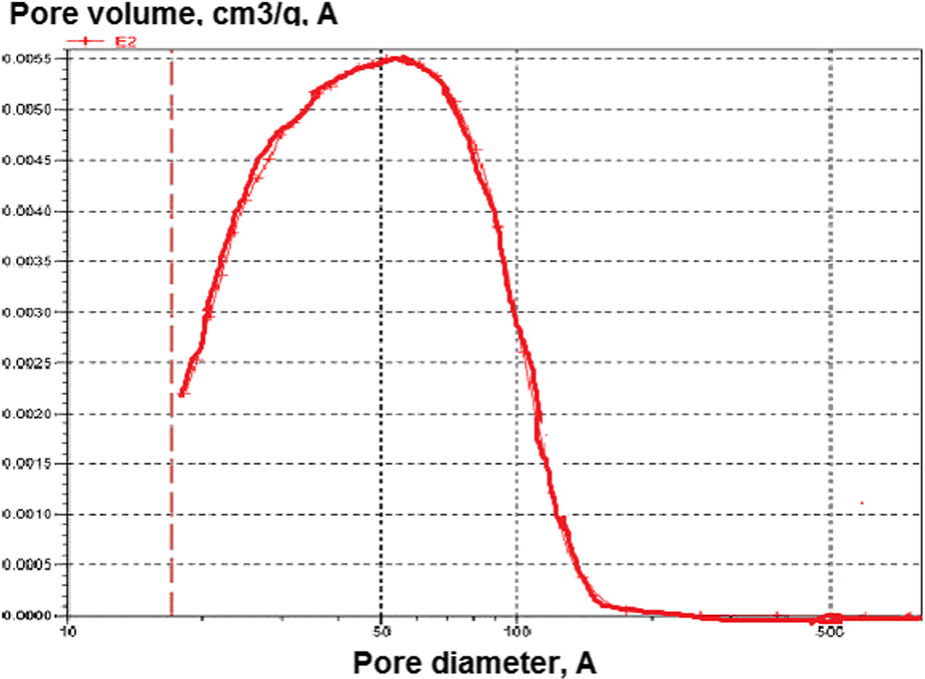
Pore size distribution curve of the composite surface

For a more detailed study of changes in the structure of the silica gel surface after modification by polymer, its surface pore size distribution diagrams were built, calculated using the BET method (Fig. 6).

From this diagram, it follows that pore size 40–60 Ǻ dominates in this composite, and the modification has virtually no effect on the pore size of the silica gel which is before and after the modification is predominantly macroporous in nature.

**Fig. 7:**
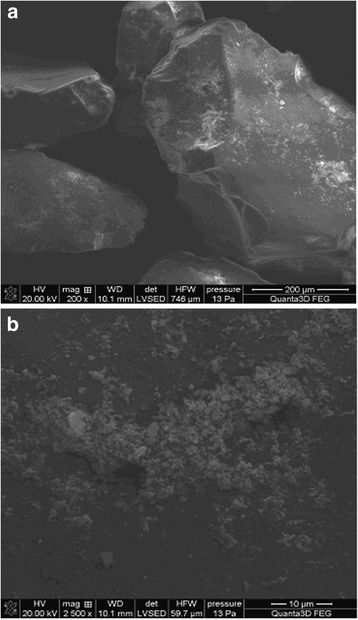
Scanning electron microscopy photo of the surface of silica gel, in situ modified by poly[*N*-(4-carboxyphenyl)methacrylamide] (**a** without grinding of the sample or **b** after grinding of a sample of silica gel)

No changes in the morphology of the silica surface after modification by the polymer are generally characteristic of “insular” or “globular” disposition of the polymer on a solid surface. This result was confirmed by scanning electron microscopy (Fig. 7).

The research results of silica gel adsorption capacity, in situ modified by poly[*N*-(4-carboxyphenyl)methacrylamide] on ions Cu(II), Pb(II), Mn(II), Fe(III), Ni(II), and Cd(II) at different values of pH and chemical composition of environment, are shown in Table 1. The analysis of this table shows that the synthesized composite detects the highest adsorption activity against microquantities:

**Table 1:** The dependence of the degree of adsorption of metal ions on silica gel with immobilized poly[*N*-(4-carboxyphenyl)methacrylamide] (terms experiment: sorbent mass—0.1 g, volume solutions—25–100 ml, *m* ^*0*^_*M*_—00 mg)

pH and chemical composition of the environment	Degree of adsorption, %					
	Cu(II)	Cd(II)	Ni(II)	Mn(II)	Pb(II)	Fe(III)
1.0 (0.1 M HCl)	21.28	6.58	63.86	6.58	59.43	5.88
1.7 (oxalate buffer)	16.83	11.05	40.05	11.05	99.99	6.08
4.0 (phthalate buffer)	15.82	1.05	65.55	1.05	55.81	33.15
5.5 (distilled water)	51.52	6.58	99.99	6.58	57.55	51.46
8.1 (sodium bicarbonate solution)	–	95.60	97.46	95.60	60.24	–
8.4 (ammonium acetate buffer)	70.20	21.32	–	21.32	–	54.07

Precipitation by hydrolysis of the salts of the corresponding metals was observed

- Ions Cu(II) and Fe(III) in a neutral environment are in the form of aqua complexes and in slightly alkaline environment, which created ammonium acetate buffer, are in the form of acetate complexes
- Ions Cu(II) and Mn(II) in a weakly alkaline environment are in the form of acetate complexes. This is an interesting fact that in sodium bicarbonate solution, microquantities of these metals are adsorbed almost completely in the presence of ammonium acetate buffer, which from the acetate complexes, adsorption is 21% only. The reason for this phenomenon can be explained as follows: a solution of sodium bicarbonate ions cadmium(II) and manganese(II) mainly exist in the form of hydroxy ions M(OH)^+^ and high adsorption compared with ammonium acetate buffer due to their partial subsidence at the surface of the modified silica gel. In the presence of ammonium acetate buffer, ions of these metals are mainly in the form of acetate complexes and adsorption in these conditions is due to the formation on the surface of mixed-ligand complexes involving acetate groups, water molecules, and the acid groups of the modified polymer.

Quantitative adsorption was fixed on ions Ni(II) in a neutral environment where ions Ni(II) are in the form of aqua complexes [Ni(H_2_O)_2_]^2+^, and on the Pb(II) ions in an acidic environment, against oxalate buffer.

In order to establish the values of the adsorption capacity of the synthesized composites on the selected transition metal ions, adsorption isotherms were built and were compared with those for the source silica gel.

**Fig. 8:**
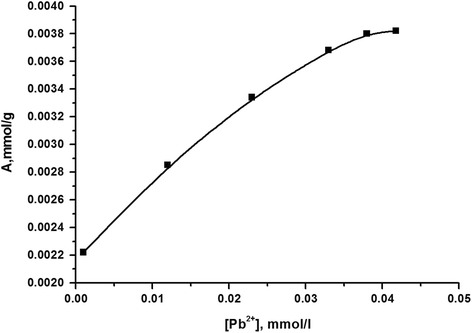
Adsorption isotherm of ions Pb(II) on silica gel with immobilized poly[*N*-(4-carboxyphenyl)methacrylamide] at pH 1.7, created oxalate

**Fig. 9:**
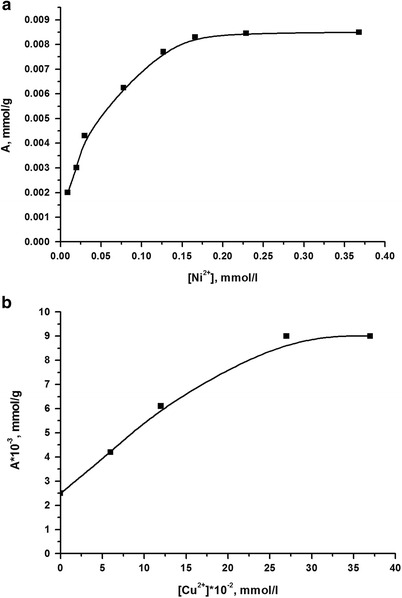
Adsorption isotherms of ions Ni(II) (**a**) and Cu(II) (**b**) on silica gel with immobilized poly[*N*-(4-carboxyphenyl)methacrylamide] from aqueous solutions of their nitrates (without addition of buffer solutions)

Examples of such isotherms for systems Pb(II), Ni(II), and Cu(II) are shown in Figs. 8 and 9. Their basis can make a preliminary conclusion that the adsorption capacity for research synthesized composite transition metal ion complexation process caused by oxygen atoms of homopolymer.

The comparison of adsorption capacity of the synthesized composite on ions of Cu(II), Pb(II), and Ni(II), calculated based on adsorption isotherms, with the source silica gel was made (Table 2). The data of this table suggest a slight increase in the adsorption capacity of silica for these ions after the modification of the surface by poly[*N*-(4-carboxyphenyl)methacrylamide], which can be explained as mainly globular placing of polymer on a solid surface, thereby creating steric difficulties accessing metal ions to most donor atoms of immobilized polymer that are inside globules [16].

**Table 2:** Adsorption capacity of the synthesized composite

Cation	Adsorption capacity			
	Source silica gel		Composite	
	mmol/g	mg/g	mmol/g	mg/g
Cu(II)	0.006	0.38	0.010	0.64
Pb(II)	0.002	0.41	0.004	0.82
Ni(II)	0.001	0.06	0.0085	5.02

## Conclusions

A new organic composite material has been synthesized by in situ immobilization of poly[*N*-(4-carboxyphenyl)methacrylamide] on the silica gel surface. The fact of heterophase polymerization has been confirmed by IR spectroscopy and mass spectrometry. As a result of thermogravimetric analysis, it has been found that the composition of synthesized composite includes 16 wt.% of polymer. Results of study options after silica surface modification of the polymer by BET and according to scanning electron microscopy show that immobilized polymer has no effect on the structure of the silica gel surface layer practically. A slight increase in the adsorption capacity of silica gel after modification on microquantities of Pb(II), Cu(II), and Ni(II) ions has been revealed.

## Abbreviations

AIBN: 2, 2'-Azobisisobutyronitrile
DTA: Differential thermal analysis
IR: Infrared
TG: Thermogravimetry
THF: Tetrahydrofuran
